# Clinical Application of Antioxidants to Improve Human Oocyte Mitochondrial Function: A Review

**DOI:** 10.3390/antiox9121197

**Published:** 2020-11-28

**Authors:** Cristina Rodríguez-Varela, Elena Labarta

**Affiliations:** 1 IVI Foundation—IIS La Fe, Fernando Abril Martorell 106, Torre A, Planta 1ª, 46026 Valencia, Spain; elena.labarta@ivirma.com; 2 IVIRMA Valencia, Plaza de la Policía Local 3, 46015 Valencia, Spain

**Keywords:** mitochondrial function, antioxidants, oxidative stress, ROS, oocyte quality, embryo quality, oxygen metabolism

## Abstract

Mitochondria produce adenosine triphosphate (ATP) while also generating high amounts of reactive oxygen species (ROS) derived from oxygen metabolism. ROS are small but highly reactive molecules that can be detrimental if unregulated. While normally functioning mitochondria produce molecules that counteract ROS production, an imbalance between the amount of ROS produced in the mitochondria and the capacity of the cell to counteract them leads to oxidative stress and ultimately to mitochondrial dysfunction. This dysfunction impairs cellular functions through reduced ATP output and/or increased oxidative stress. Mitochondrial dysfunction may also lead to poor oocyte quality and embryo development, ultimately affecting pregnancy outcomes. Improving mitochondrial function through antioxidant supplementation may enhance reproductive performance. Recent studies suggest that antioxidants may treat infertility by restoring mitochondrial function and promoting mitochondrial biogenesis. However, further randomized, controlled trials are needed to determine their clinical efficacy. In this review, we discuss the use of resveratrol, coenzyme-Q10, melatonin, folic acid, and several vitamins as antioxidant treatments to improve human oocyte and embryo quality, focusing on the mitochondria as their main hypothetical target. However, this mechanism of action has not yet been demonstrated in the human oocyte, which highlights the need for further studies in this field.

## 1. Introduction

**Figure 1:**
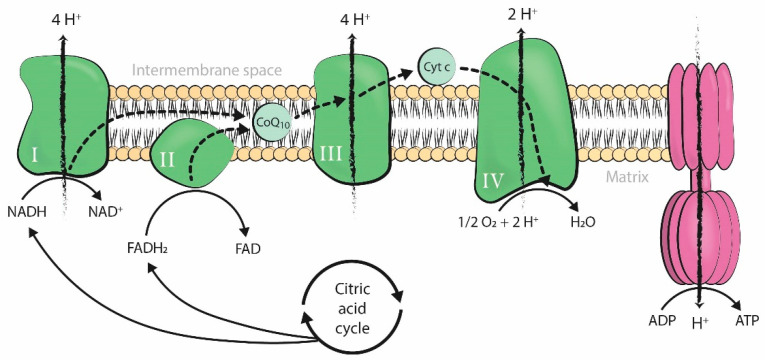
Production of adenosine triphosphate (ATP) by oxidative phosphorylation coupled to the mitochondrial electron transport chain. The four enzymatic complexes (I, II, III, and IV) and ATP synthase are represented in the inner mitochondrial membrane. ADP: adenosine diphosphate; ATP: adenosine triphosphate; Pi: inorganic phosphate; H+: hydrogen ion (proton); NADH: nicotinamide adenine dinucleotide, reduced form; FADH_2_: flavin adenine dinucleotide, reduced form; NAD+: nicotinamide adenine dinucleotide, oxidized form; FAD: flavin adenine dinucleotide, oxidized form; O_2_: oxygen; H_2_O: water; Cyt c: cytochrome c; CoQ10: coenzyme-Q10.

Mitochondria produce the energy required by cells to carry out all cellular processes. Energy is generated in the form of adenosine triphosphate (ATP) through oxidative phosphorylation, a process that takes place in the inner mitochondrial membrane under aerobic conditions. Along this membrane, electrons from the controlled oxidation of nicotinamide adenine dinucleotide (NADH) or flavin adenine dinucleotide (FADH_2_), both products of the citric acid cycle, travel through several enzymatic complexes forming the electron transport chain (ETC). The movement of electrons throughout the ETC is coupled with the transfer of protons across the membrane into the intermembrane space, generating an electrochemical proton gradient over the inner mitochondrial membrane that is harnessed by F1-F0 ATPase to phosphorylate adenosine diphosphate (ADP) into ATP [1] (Figure 1).

Mitochondrial respiration is a form of aerobic metabolism and uses oxygen to produce energy, with oxygen as the ultimate electron acceptor of the electron flow system of the mitochondrial ETC. However, mitochondrial electron flow may become uncoupled at several sites along the chain, resulting in unpaired single electrons that react with oxygen or other electron acceptors and generate free radicals. When these electrons react with oxygen, the resulting free radicals are referred to as reactive oxygen species (ROS). These include the superoxide anion (O_2_^•−^), which forms hydrogen peroxide (H_2_O_2_) and can further react to form the hydroxyl radical (HO^•^). Unrelated to respiration, there is also a large source of H_2_O_2_ in the outer mitochondrial membrane due to monoamine oxidase catalytic activity [1,2].

Physiological levels of ROS are required for normal cellular function [3]. However, ROS are also highly reactive molecules that can damage mitochondrial components, initiate degradative processes, deregulate essential cellular functions, and initiate many pathological conditions if generated uncontrollably [1]. Therefore, many organisms have developed a system of antioxidant defense, in which mitochondria play a major role as antioxidant producers, allowing them to maintain balanced levels of oxidants and antioxidants [3]. An antioxidant is any substance that delays the oxidation of lipids, carbohydrates, proteins, or DNA by directly scavenging ROS or by indirectly up-regulating antioxidant defenses or inhibiting ROS production. There are many different endogenous and exogenous sources of antioxidants [3,4], but the first line of defense is also the main ROS producer in the cell: the mitochondria.

Antioxidants counteract the high levels of ROS derived from mitochondrial metabolism, reducing damage to the cell. However, an imbalance between the amount of antioxidants and ROS produced, in favor of the latter, leads to oxidative stress [3]. Oxidative stress generates lipid peroxidation [5], as well as RNA, DNA, and protein oxidation, which in turn leads to their selective enzymatic degradation by nucleases and proteases [2]. On the one hand, lipid peroxidation affects the integrity of cell membranes [5]. On the other hand, nuclear DNA degradation induces the onset of apoptosis [6] and occurs at the same time as the release of mitochondrial cytochrome c (Cyt c) [2], which is also responsible for the initiation of programmed cell death [7]. Oxidative stress may also interfere with essential mitochondrial functions within the cell by promoting the inactivation of enzymes from the mitochondrial ETC [8] and by increasing mtDNA mutations. In fact, mitochondrial DNA is prone to mutations because it lacks protective histones and is in close proximity to the inner mitochondrial membrane [9]. Finally, oxidative stress has also been related to telomere shortening and senescence [10].

Oxidative stress can be caused by, or be the cause of, mitochondrial dysfunction (MD). MD reduces the production of ATP and synthesis of antioxidant molecules, creating a cycle in which ROS-induced mitochondrial damage results in higher oxidant production and further mitochondrial impairment [11]. MD is involved in the pathogenesis of many neurodegenerative and cardiovascular diseases, such as Alzheimer’s disease and atherosclerosis [12,13]. In the reproductive field, MD is related to a decline in oocyte quality [14]. Mitochondria are essential organelles involved in meiotic spindle assembly, proper segregation of chromosomes, maturation, fertilization, and embryo development [15]. Therefore, MD may affect the quality and DNA content of oocytes, embryo development, and pregnancy outcome. The consequences of MD are not limited to the short-term, as oxidative stress exposure during the gestational period is related to long-lasting cardiovascular effects [16].

Regardless of origin, oxidative stress and MD are triggered by both intrinsic and extrinsic factors. Intrinsic factors include biological age [11,17], endometriosis [18], polycystic ovarian syndrome (PCOS) [19], and premature ovarian insufficiency (POI) [20]. Extrinsic factors include environmental exposure to ROS inducers or producers, such as diet, professional exposure, and assisted reproduction treatment (ART) techniques [21]. Some of these factors are modifiable and, therefore, offer opportunities for intervention.

ROS are natural products of sperm, oocyte, and embryo metabolism. However, gamete manipulation during ART procedures increases ROS either through indirect intracellular ROS production in response to external stressors or through direct exogenous ROS production by environmental factors. The risk of oxidative stress development is higher in vitro than in vivo, although it remains unclear to what extent ART is responsible for higher levels of oxidative stress [21]. Despite recent advancements in ART techniques [22,23,24,25], the in vitro fertilization (IVF) setting does not recreate the conditions of natural fertilization, which includes tight physiological regulation of oxidative stress by antioxidants. Oxygen concentration, temperature variation, high light exposure, culture media composition, and cryopreservation methods are environmental sources of oxidative stress in the IVF laboratory [21], implicating the need for antioxidant supplementation in the IVF setting. Indeed, human IVF culture media are supplemented with combinations of molecules with antioxidant properties, including human serum albumin, ethylenediaminetetraacetic acid, folic acid, ascorbic acid, and pantothenic acid (vitamin B5), among others [25] (see Supplemental Data).

**Figure 2:**
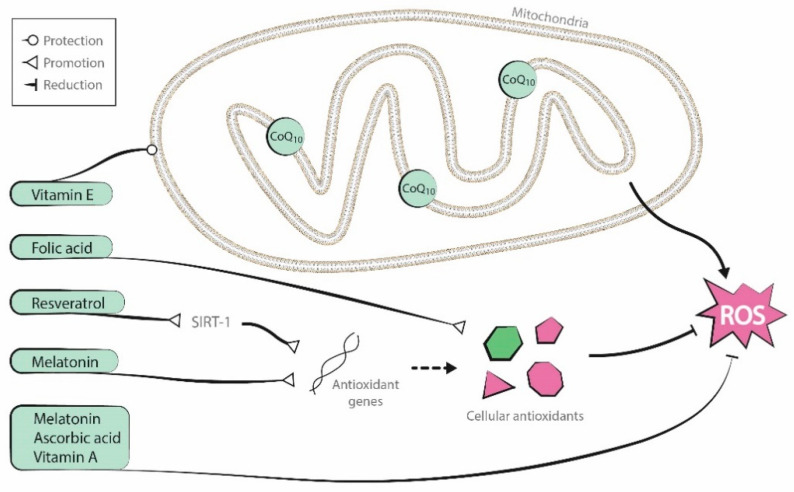
Antioxidant properties of the molecules reviewed (coenzyme-Q10, resveratrol, melatonin, and vitamins A, B, C, and E).

Antioxidants are endogenous to organisms, but it is uncertain if supplementation of these substances can improve oocyte mitochondrial function. Although several supplementary antioxidant molecules have shown promising results [26,27,28], two recent Cochrane reviews described low-quality evidence about the positive effects of oral antioxidant treatment in live birth and clinical pregnancy rates in women attending an infertility clinic [29,30]. In this review, we describe the effects of several antioxidant supplementation treatments in human oocytes and embryos. The antioxidants discussed include coenzyme-Q10, resveratrol, melatonin, and vitamins A, B (folic acid), C (ascorbic acid), D, and E. Figure 2 presents a graphic representation of their antioxidant properties.

## 2. Antioxidant Supplementation in Reproduction

Antioxidant treatment in the reproductive field can be carried out either by oral supplementation before infertility treatment or by culture media supplementation during ART. Oral supplementation attempts to improve gamete quality in vivo, while culture media supplementation attempts to do so in vitro. The latter approach can also be used to improve the in vitro maturation (IVM) process and to counteract high ROS production within the IVF setting. 

Antioxidant supplementation is generally described in the literature as being applied to the male [31]. In this review, we discuss the use of antioxidants to improve oocyte and embryo quality, both in vivo and in vitro. We focus on mitochondrial function because its enhancement may be the main mechanism by which antioxidants manage to improve gamete quality. However, although mitochondrial function has been restored by different antioxidant molecules in many other tissues [32,33,34,35], this direct association has not yet been demonstrated in the human oocyte. A summary of the main evidence regarding the current utility of each of the antioxidants described is presented in Table 1, while a more extensive summary of the results of the discussed human studies is presented in Table 2.

### 2.1. Coenzyme-Q10

Coenzyme-Q10 (CoQ10) carries electrons from complexes I and II to complex III in the mitochondrial respiratory chain and participates in the synthesis of ATP [36] (Figure 1). CoQ10 is a source of superoxide anion radical, though it also acts as an antioxidant, making it both a prooxidant and an antioxidant. The reduced form of CoQ10, ubiquinol, protects biological membranes from lipid peroxidation by recycling vitamin E and is also an antioxidant [37].

The dual role of CoQ10 in controlling mitochondrial function makes it an essential molecule for cellular performance. CoQ10 plasma levels decrease with advancing age, and this decline coincides with a decline in fertility and an increase in embryo aneuploidies [38]. Supplementation with CoQ10 may improve the reproductive outcome in infertile patients by improving mitochondrial function. Indeed, preovulatory CoQ10 supplementation improves mitochondrial function in aged mice [39] and improves non-aging related MD in mice [40] and other species [41]. In addition, CoQ10 treatment prevented mitochondrial ovarian aging in a rat model [42]. CoQ10 supplementation in culture media also restored the age-induced deterioration of oocyte quality in mice [43] and porcine models [44]. Finally, CoQ10 injected into aged mice increased mitochondrial respiratory activity and glucose uptake in cumulus cells, as well as the number of cumulus cells per oocyte, leading to improved reproductive capacity. These findings suggest that CoQ10 supplementation can benefit not only oocytes but also cumulus cells; this can be translated to humans because the human aging process also leads to reduced CoQ10-synthesis gene expression in cumulus cells [45].

#### Use of Coenzyme-Q10 in Infertility

In humans, higher CoQ10 levels in follicular fluid are related to improved embryo quality and higher pregnancy rates [46]. However, although CoQ10 supplementation increases CoQ10 levels in follicular fluid in infertile patients [47], clinical outcome results are inconclusive. CoQ10 supplementation (600 mg/day for 2 months and up to the day of oocyte retrieval) in women with infertility of advanced age (between 35 and 43 years old) was insufficient to prevent ROS prolonged exposure effects on the meiotic apparatus because there was no significant difference in aneuploidy rates between the treated and the control groups. However, the study ended prematurely due to concerns related to the possible deleterious effects of polar body biopsy on embryo quality and implantation. The study did not recruit the number of participants initially proposed, which could underlie the non-significant difference in aneuploidy rate (46.5% CoQ10 group vs. 62.8% control group) [26]. Moreover, the dose and duration of CoQ10 treatment were based on mice studies, so humans may require longer exposure or higher doses to achieve remarkable benefits [26]. 

Similarly, CoQ10 supplementation (600 mg/day for 2 months before ovarian stimulation) increased ovarian response [mean (interquartile range, IQR); 4 (2,5) vs. 2 (1,4) oocytes; *p* = 0.002], fertilization rates (67.5% vs. 45.1%; *p* = 0.001), and the number of high-quality embryos [1 (0,2) vs. 0 (0,1.75); *p* = 0.03] in young women with poor ovarian reserve. However, no differences in clinical pregnancy, miscarriage, or live birth rates were observed [48]. A recent systematic review and meta-analysis of five randomized controlled trials (RCTs) indicated that CoQ10 oral supplementation, as an IVF pre-treatment, increased clinical pregnancy rates (CPR) in comparison with a placebo or no treatment [28.8% vs. 14.1%; odds ratio (OR) 2.44, 95% confidence interval (CI) 1.30–4.59, *p* = 0.006]. However, characteristics of the study population, as well as the dose and duration of CoQ10 treatment, were highly heterogeneous between the studies analyzed. No differences were observed in live birth rates (LBRs) (28% vs. 17.4%; OR 1.67, 95% CI 0.66—4.25, *p* = 0.28), or miscarriage rates (MR) (10% vs. 13.6%; OR 0.61, 95% CI 0.14–2.76, *p* = 0.52, in treated and untreated women, respectively). The lack of statistical power may be explained by insufficient data regarding these two variables [49]. 

Additionally, CoQ10 supplementation benefited PCOS patients who exhibited ovulatory disorders and a higher proportion of immature follicles within the ovary [19]. Indeed, an RCT showed a significant increase in the number of follicles ≥14 and 18 mm of clomiphene citrate (CC)-resistant PCOS patients after the addition of CoQ10 as an adjuvant to CC treatment [50]. 

CoQ10 supplementation is proposed as an adjuvant during IVM, which may promote this technique by improving mitochondrial function in immature oocytes. Despite the initial discrepancies between animal studies [51,52], a 2020 report by Ma et al. described increased maturation rates (82.6% vs. 63.0%; *p* = 0.035) and reduced post-meiotic aneuploidies (36.8% vs. 65.5%; *p* = 0.02) after IVM in oocytes from women of advanced age supplemented with CoQ10. They did not find significant differences in young women [53]. However, mitoquinol (a CoQ10 analog) addition to the culture media from fertilization until the blastocyst stage did not improve embryo quality in women of advanced age. There were no significant differences in day 5 (18% vs. 20%) or total (48% vs. 45%) good quality blastocyst development per zygote, total blastocyst development (63% vs. 62%), and euploidy rates (33% vs. 30%) between the control and the treatment groups, respectively [54]. 

In sum, although CoQ10 is a promising therapy and is non-pharmaceutical, inexpensive, and safe, there is a need for well-designed clinical trials involving a greater number of patients to fully assess the effects of CoQ10 treatment on human fertility.

### 2.2. Resveratrol

Resveratrol is a natural polyphenol synthetized by several plants in response to pathogens. It is found in grapes, red wine, peanuts, and several medicinal plants [55]. Over the past decade, resveratrol has emerged as a therapeutic treatment for many diseases due to its anti-aging, antioxidant, anti-inflammatory, insulin-sensitizing, cardioprotective, vasodilating, and anti-neoplastic properties [56]. In the reproductive field, resveratrol may benefit women with diminished ovarian function, PCOS, endometriosis, and uterine fibroids [55,57,58,59]. Resveratrol may improve age-related decline in ovarian function through the activation of sirtuin 1 (SIRT1) [60], a molecule that protects mitochondrial function from oxidative stress and whose levels are undetectable in aged oocytes [61]. In rats, resveratrol supplementation inhibited the process of follicular atresia, increased ovarian follicular reserve, and prolonged ovarian lifespan [62]. Moreover, mice treated with resveratrol for 12 months exhibited a higher number of follicles than controls and improved the number and quality of oocytes, evidenced by proper spindle morphology and chromosome alignment. In addition, telomerase activity, telomerase length, and age-related gene expression in the ovaries of mice supplemented with resveratrol resembled those of younger mice [63]. Resveratrol may also improve ovarian dysfunction caused by POI through the inhibition of the PI3K/AKT [64] and the NF-kB signaling pathways [65]. Resveratrol inhibited oxidative stress and inflammatory events in a rat POI model [66], and its antiapoptotic effect prevented oogonial stem cell loss in a mouse model of POI [67]. Furthermore, the addition of resveratrol to culture media improved oocyte maturation and embryo developmental potential in different animal studies, both in IVM [68,69] and conventional in vitro culture [70], by decreasing ROS production and increasing ATP content. Resveratrol may enhance mitochondrial homeostasis in both oocytes and granulosa cells via SIRT1 activation and regulation of the balance between mitochondrial biogenesis and autophagy [69]. Resveratrol may also have an antiapoptotic effect on granulosa cells through the inhibition of NF-kB signaling [65]. Nevertheless, the effects of resveratrol depend largely on cell type [55]. Contrary to granulosa cells, resveratrol exerts a proapoptotic effect on theca cells and counteracts insulin’s stimulatory effect on cell proliferation [71]. In addition, resveratrol inhibits theca-interstitial cell androgen production, primarily by inhibiting the rate-limiting enzyme in the androgen biosynthesis pathway [72]. Therefore, it is useful in PCOS treatment, a pathology related to insulin resistance/hyperinsulinemia, theca-interstitial cell hyperplasia, and hyperandrogenism [55]. In vivo studies have demonstrated the antioxidant effect of resveratrol and its ability to return ovarian morphology to normal limits in a PCOS rat model [73]. 

In the endometrium, resveratrol’s antiapoptotic and anti-proliferative effects inhibited the progression of ectopic endometrium, countering endometriosis [58]. In addition, resveratrol reduced vascular endothelial growth factor (VEGF) expression [74], improving the treatment of ovarian hyperstimulation syndrome (OHSS) and endometriosis, both gynecologic disorders associated with excessive VEGF activity [55]. However, its anti-inflammatory properties may inhibit the inflammatory-related process of decidualization [75], leading to decreased endometrial receptivity. 

**Figure 3:**
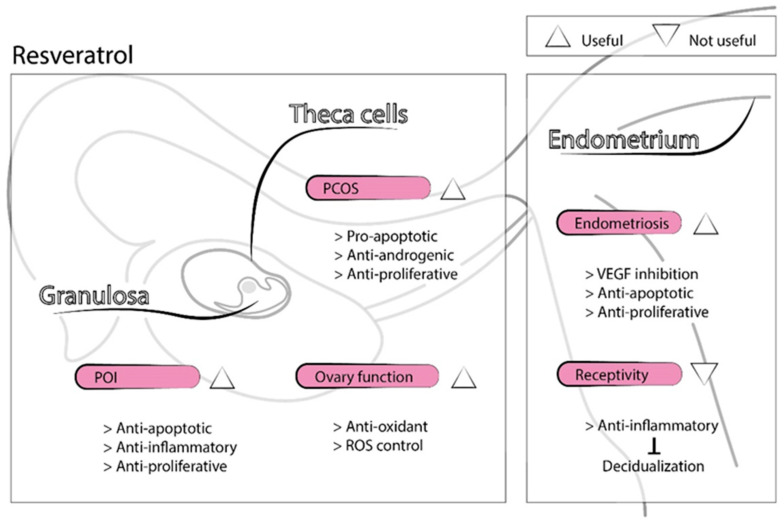
Representation of resveratrol’s physiological functions in the reproductive field.

Therefore, resveratrol has the potential to benefit women with diminished ovarian reserve and function through its antioxidant effects and the stimulation of mitochondrial biogenesis, though it also has adverse effects on implantation and endometrial decidualization [76] (Figure 3). Human studies involving resveratrol are needed to validate the success observed in animal models. 

#### Use of Resveratrol in Infertility

In humans, resveratrol supplementation in the IVM medium of aged immature oocytes demonstrated improved oocyte maturation rates after 24 h (55.3% vs. 37.84%; *p* < 0.05) and 36 h of culture (71.1% vs. 51.35% in the study and control group, respectively; *p* < 0.05). Resveratrol also improved oocyte quality, as evidenced by improved mitochondrial immunofluorescence intensity (53.0% vs. 31.1%, *p* < 0.05) and a reduced proportion of oocytes with abnormal spindle morphology and irregular chromosomal disposition (*p* < 0.05) [68]. In a double-blind RCT, oral resveratrol (1500 mg/day) supplementation in women with PCOS showed reduced ovarian and adrenal androgen levels [77]. A triple-blinded RCT comparing 800 mg/day of resveratrol to a placebo in patients with PCOS found an increased high-quality oocyte rate (81.9% vs. 69.1%; *p* = 0.002), increased high-quality embryo rate (89.8% vs. 78.8%; *p* = 0.024), and reduced expression of the VEGF gene in granulosa cells (*p* = 0.0001) [78]. Finally, a retrospective study evaluated the impact of resveratrol (200 mg/day) on human pregnancy outcomes during fresh or frozen embryo transfer cycles compared to no treatment. The study group showed significantly lower clinical pregnancy rates (10.8% vs. 21.5%; *p* = 0.0005), as well as higher miscarriage rates (52.4% vs. 21.8%; *p* = 0.0022), even after a multivariate logistic regression analysis (CPR: OR 0.539, 95% CI 0.341–0.853; MR: OR 2.602, 95% CI 1.070–6.325). Patients with resveratrol treatment had poor pregnancy outcomes, even though good quality embryos had been transferred [27], which may be related to the suppressor effect of resveratrol on decidualization [75]. This study focused on pregnancy outcomes after embryo transfer and did not evaluate ovarian function before or after resveratrol treatment [76].

Following these results, an alternative resveratrol administration protocol was proposed to avoid negative effects on the endometrium. Because the half-life of resveratrol is between 3 and 9 h [79], it may not affect implantation or pregnancy if its endometrial tissue levels drop before decidualization. Thus, discontinuation of resveratrol intake on the day of ovulation, or the freeze-all policy with a deferred frozen embryo transfer without supplementation, may help overcome these adverse effects [76]. 

Resveratrol may have adverse effects depending on the dose administered. Some side effects were reported after its administration, including headaches, nausea, diarrhea, dizziness, and liver dysfunction [79]. High-dose resveratrol should not be administered, and its supplementation should be discontinued during pregnancy due to its negative effect on the endometrial decidualization process and scarce data about adverse effects and embryo-fetal toxicity. Randomized controlled trials are needed along with human studies to define a standard dose and duration of treatment since there is a high heterogeneity between all the studies described [55].

### 2.3. Melatonin

Melatonin is synthesized from the amino acid tryptophan, almost exclusively by the pineal gland. It is released into the blood in a circadian manner, and its production is restricted to nighttime [80]. Melatonin may be produced in high amounts in mitochondria [34], and it has an important role in reducing oxidative stress. Its antioxidant properties come from its excellent free radical scavenging, as well as its capacity to upregulate the expression of antioxidant enzymes and a spectrum of stress-responsive genes. In addition, melatonin indirectly accelerates electron transport through the ETC, decreasing electron leakage and reducing ROS formation, a process called radical avoidance [81]. Moreover, this molecule has antiapoptotic [82], anti-inflammatory [83], and antiandrogenic properties [84]. 

Melatonin modulates the hypothalamic–pituitary–gonadal axis at different levels, thus modulating reproductive system activity [85]. Melatonin may reduce intrafollicular oxidative stress, and melatonin levels in follicular fluid are suggested as a biomarker to predict IVF outcomes and ovarian reserve [86]. Melatonin production decreases with age [87], and in the ovary, this age-related decline is related to the increase in follicle-stimulating hormone levels associated with menopause [88]. Therefore, melatonin may delay ovarian aging. Interestingly, Song et al. found reduced aging ovary parameters, increased oocyte quality and quantity, and increased litter size in mice treated with melatonin for 12 months [89].

#### Use of Melatonin in Infertility

In humans, melatonin treatment improves fertility outcomes in infertile women [28,90,91,92], patients with PCOS [84], patients with endometriosis, and many other gynecologic pathologies. In addition, melatonin may help normalize menstrual disorders, such as dysmenorrhea [93]. Further, melatonin supplementation during pregnancy may protect the embryo from oxidative stress since this hormone crosses the placental barrier during pregnancy [94].

Melatonin supplementation is proposed as a treatment for poor oocyte quality due to its antioxidant effect in oocytes and granulosa cells. In 2003, Takasaki et al. found that melatonin treatment (3 mg/day from the previous cycle until the day of triggering) improved oocyte quality in women with a previous IVF failure due to poor oocyte quality. However, this improvement was evidenced only in the number of degenerated oocytes retrieved and not in the number of oocytes or mature oocytes obtained. In the treatment group, melatonin levels were significantly higher in the follicular fluid, while levels of lipid peroxide were reduced [90]. Lipid peroxide is implicated in free radical reactions [5]. Furthermore, melatonin accelerates the action of the maturation-inducing hormone, a molecule important to final oocyte maturation [95]. Thus, melatonin may improve oocyte quality by protecting oocytes from oxidative stress and enhancing the oocyte maturation processes. 

Clinical trials found that melatonin supplementation during an IVF cycle significantly increased the number of retrieved (11.5 vs. 6.9 in the control group; *p* = 0.0001) and mature oocytes (9.0 vs. 4.4 in the control group; *p* = 0.0001) [28], or at least the proportion of metaphase II (MII)/retrieved oocytes (81.9% vs. 75.8% in the control group; *p* = 0.034) [92]. In addition, the fertilization success of women with low fertilization rates (≤50%) in the previous IVF cycle was improved by melatonin treatment compared to the previous cycle (29.8% increment in the melatonin group vs. 1.9% increment in the non-melatonin group; *p* < 0.01) [91]. However, an RCT conducted in 2018 found no difference in the number of oocytes retrieved, number of MII, fertilization rate, embryo quality, clinical pregnancy rate, or live birth rate between the treatment and control groups [96]. 

In 2019, Espino et al. found reduced concentrations of melatonin at the systemic level and in the follicular fluid of women with unexplained infertility. Interestingly, melatonin levels were correlated with different parameters of oxidative balance in follicular fluid. In comparison with a control group of fertile women, patients with unexplained infertility who underwent melatonin supplementation during ovarian stimulation until the day of oocyte retrieval (one group 3 mg/day and the other group 6 mg/day) had better outcomes than patients with no melatonin treatment. This supplementation restored concentrations of diverse oxidative stress markers, improved oocyte quality, and, consequently, increased the number of transferable embryos and the success of ART [97]. Lastly, melatonin supplementation of culture media also improved outcomes in animal studies in IVM [98] and conventional in vitro culture [99]. IVM of immature oocytes from PCOS patients cultured with melatonin achieved elevated but not significantly higher maturation rates [100]. 

The main advantage of melatonin antioxidant therapy is its relatively well-documented safety due to its frequent use as a sleep aid [101]. However, studies conducted so far have evaluated melatonin only in the short-term, so long-term clinical trials are needed [102]. 

### 2.4. Vitamins

Despite their proven antioxidant properties, the use of vitamins as supplements to improve mitochondrial oocyte function has not been extensively addressed. No human clinical trials have assessed the role of vitamins as antioxidants in improving oocyte and embryo quality in IVF patients. 

#### 2.4.1. Vitamin A

Vitamin A is a lipid-soluble vitamin obtained from the diet in the form of vitamin A esters or provitamin A carotenoids. Dietary sources of vitamin A include green and yellow vegetables, dairy products, fish, eggs, and organ meats. There are at least a dozen forms of vitamin A esters and approximately 600 types of carotenoids, although only about 50 forms of the latter have provitamin A activity. Once within the body, physiologically active forms of vitamin A are retinol, retinal, and retinoic acid [103]. 

Antioxidant activity has been described for retinol, the main physiological form of vitamin A, as well as for α and β carotenes, two examples of provitamin A carotenoids. Vitamin A forms are potent antioxidants that act by scavenging peroxyl radicals, thus reducing ROS levels [103]. In the reproductive field, vitamin A and its metabolites have important roles in follicular growth [104], steroidogenesis [105], oocyte maturation [106,107], and embryo development [108]. Its supplementation has been tested in both in vivo [109,110] and in vitro studies. In vitro studies found that vitamin A addition to IVM medium increased maturation rates by enhancing mitochondrial membrane potential activity, lowering ROS levels, and decreasing apoptosis [111]. Indeed, retinoic acid supplementation to the IVM medium has obtained beneficial results in several species, including cows [112] and mice [113].

##### Use of Vitamin A in Infertility

In humans, high follicular fluid levels of all-trans retinoic acid (ATRA), the active metabolite of retinol, at the time of oocyte retrieval, were related to oocytes giving rise to embryos of the highest quality. In addition, follicular fluid ATRA concentrations were positively correlated with patient serum ATRA [114]. A later study from the same group confirmed that this higher oocyte competence was the result of higher ATRA synthesis of cumulus-oocyte complexes, which in turn was positively correlated with higher fertilization rates [115].

Therefore, minimum levels of vitamin A or its metabolites are essential for proper oocyte maturation and acquisition of competence. However, due to the essential role of vitamin A in the signaling pathways that control ovarian function, including oocyte maturation and development [116], it is unknown if its antioxidant properties also play a role in these processes. In any case, human clinical trials assessing the effect of vitamin A supplementation in oocyte quality have not been performed yet, neither in vitro nor in vivo.

#### 2.4.2. Folic Acid

Folate, or vitamin B9, is a B vitamin found in green leafy vegetables, dark green vegetables, beans, and other legumes. The synthetic form of folate, folic acid, is common in dietary supplements in fortified foods due to its high stability and low cost [117]. At the cellular level, folate acts as a methyl donor to support the methylation of homocysteine to become methionine [118], which is a critical intermediary in the production of glutathione, the primary intracellular antioxidant. Therefore, folic acid may protect from oxidative stress by increasing intrinsic antioxidant levels within the cell. In addition, different molecules derived from folate metabolism are also essential to DNA, RNA, protein synthesis, proper epigenetic activity, and chromosome structure maintenance, making folate indispensable during periods of rapid cell growth and proliferation, such as germ cell maturation, pregnancy, and fertilization [119]. 

In Europe, the recommended folate intake in adult women ranges from 170 to 300 µg/day, and 400 µg/day of supplemental folic acid is recommended for pregnant women [120]. Folate deficiency can occur due to poor dietary intake, either of folate itself or of the micronutrients necessary for its synthesis, and/or malabsorption, mainly by defects in folate-metabolizing genes. Folate deficiency leads to homocysteine accumulation [121] or hyperhomocysteinemia, which is associated with several pathophysiological mechanisms during pregnancy, including oxidative stress [122] and decreased cellular antioxidant potential [123]. 

Preconception folate deficiency hampered female fertility, as well as embryo and fetal viability, in several animal models. This condition partially inhibits ovulation in rats [124] and increases the number of degenerated follicles in rhesus monkeys [125], suggesting the essential role of folate in folliculogenesis [119]. In humans, women with folate deficiency undergoing ovarian stimulation often have impaired ovarian function, lower oocyte quality, and lower pregnancy rates [126,127]. However, no human studies have hypothesized a relationship between folate deficiency-related mitochondrial impairment and reduced fertility. 

Folic acid supplementation, therefore, may be useful in the reproductive field. Its use during pregnancy is widespread, as it seems to prevent fetal neural tube defects [128] as well as heart defects [129], Down syndrome [130], intrauterine growth restriction, and pre-term birth [131]. However, its preconception use is less studied. Recently, the impact of folic acid supplementation was investigated regarding the earlier stages of female reproductive physiology, in particular its role in folliculogenesis [119]. During the preovulatory stage, folic acid decreased the proportion of developmentally delayed embryos in a mouse model [132] and increased glutathione synthesis at the oocyte level in a rat model, although it was unable to revert the altered expression pattern of genes related to mitochondrial function and dynamics [133].

##### Use of Folic Acid in Infertility

In humans, folate and homocysteine are present in the follicular fluid [134], and these levels correlate with their blood concentrations [135]. Moreover, folic acid supplementation increases serum folate concentration and reduces serum hyperhomocysteinemia [135], exerting the same effect at the follicular level. A negative correlation was found between homocysteine levels in the follicular fluid and oocyte maturity [127], as well as in vitro day-3 embryo quality [136]. Similar results were obtained in a recent study conducted in PCOS patients, where negative correlations between homocysteine concentrations and fertilization rates, as well as oocyte and embryo quality, were observed [137]. Furthermore, higher folate intake was related to higher implantation, clinical pregnancy, and live birth rates [138].

Supplemental folic acid may improve ART outcomes and is favored over food-based sources due to lower amounts of bioavailable folate in food [139]. However, literature evaluating the impact of folate levels on reproduction analyze the effects of different concentrations within the normal dietary intake range, and folic acid supplementation studies are scarce. A prospective study conducted in women with unexplained infertility found no difference in clinical pregnancy rates (32.8% users vs. 35.7% non-users) and live birth rates (24.1% users vs. 31.0% non-users) after folic acid supplementation, even though folate blood levels were increased [140].

Therefore, a diet rich in folate is essential to achieve good pregnancy outcomes. Its antioxidant properties may have an important impact in folliculogenesis and early embryo development, although folate has multiple other functions that may also assist pregnancy. However, there are no clinical trials confirming its therapeutic advantages as an antioxidant supplement to benefit infertility treatment or its potential to enhance oocyte mitochondrial function.

#### 2.4.3. Ascorbic Acid

Ascorbic acid, also known as vitamin C, is a powerful antioxidant that scavenges free radicals [141]. Its addition to culture and vitrification/warming media significantly improves the quality and survival rates of porcine cryopreserved embryos by regulating some crucial genes implicated in mitochondrial redox status [142]. Despite this, ascorbic acid supplementation to fertilization and conventional culture media did not exert any significant beneficial effect on maturation, fertilization, or embryo development parameters [143].

##### Use of Ascorbic Acid in Infertility

In humans, ascorbic acid supplementation significantly increased serum and follicular fluid ascorbic acid levels [144,145,146]. However, no differences in implantation or clinical pregnancy rates were found after ascorbic acid supplementation in women undergoing an IVF procedure, either during hormonal ovarian stimulation [144] or during the luteal phase [145]. In addition, this therapy was incapable of reducing oxidative stress markers in women with endometriosis after two months of treatment [146]. Therefore, although promising, vitamin C supplementation in infertile patients has yet to show a beneficial effect on fertility. Further clinical trials are needed to confirm preliminary results. 

#### 2.4.4. Vitamin D

Vitamin D plays a crucial role in dietary calcium absorption. In the reproductive field, vitamin D deficiency has been suggested to impact reproductive performance [147], but the evidence is not conclusive. Its potential antioxidant effect in the human female gamete has not been investigated. Currently, an RCT is being conducted in which follicular fluid and cumulus cells samples will be processed in order to evaluate the effect of vitamin D supplementation on oocyte quality, although the main primary endpoint is the clinical pregnancy rate [148]. Because vitamin D shows antioxidant properties [149] and the ability to improve mitochondrial function in other tissues [150], it could become an antioxidant treatment in the future, although its implications as an antioxidant at the oocyte molecular level need to be elucidated. 

#### 2.4.5. Vitamin E

Vitamin E is an essential antioxidant mainly found in high-fat vegetable products, and α–tocopherol is its most common form [151]. Its main role is the protection of cell membranes from oxidative damage by reaction with lipid radicals produced in the lipid peroxidation chain reaction [5]. 

Culture media supplementation with vitamin E increased the blastocyst development rate in a bovine model, presumably by protecting from ROS [152]. Similar results were found in a mouse model, although with less benefit compared to vitamin C supplementation [153]. Interestingly, combined oral supplementation with vitamins C and E successfully prevented ovarian aging in a mouse model [154].

##### Use of Vitamin E in Infertility

Bahadori et al. described several vitamin E concentration intervals related to higher human oocyte and embryo quality. Follicular fluid vitamin E levels in the ranges 0.35–1 mg/dL and 1.5–2 mg/dL were related to higher oocyte maturation rates (89.2% and 84.9%, respectively, vs. 69.6% in 1–1.5 mg/dL and 76.7% in 2–7.4 mg/dL; *p* = 0.002) while serum vitamin E levels in the range 10–15 mg/dL were related to a higher proportion of high-quality embryos (87.5% vs. 46.2 in 1–5 mg/dL, 54.9% in 5–10 mg/dL, 42.9% in 15–20 mg/dL; *p* = 0.007). However, no significant relationship between serum vitamin E levels and oocyte maturation was found, nor was there a correlation between follicular fluid vitamin E levels and embryo quality [155]. 

A recent RCT showed that the concomitant administration of vitamin E and vitamin D to women with PCOS was associated with higher implantation (35.1% vs. 8.6%; *p* < 0.001), pregnancy (69.0% vs. 25.8%; *p* < 0.001), and clinical pregnancy rates (62.1% vs. 22.6%; *p* = 0.002) compared to a control group [156]. However, these outcomes were not associated with an antioxidant mechanism. Therefore, the findings of this trial do not support the use of vitamins D and E as a dual antioxidant treatment in IVF, and further research is needed to determine whether the antioxidant mechanism of vitamin E can improve mitochondrial oocyte function.

### 2.5. Antioxidants in Combination

Antioxidants can be supplied alone or in combination, and different cocktails of these molecules were evaluated for their potential to improve oocyte quality both in animal [157,158] and human studies [159,160]. Providing antioxidants in combination may more closely resemble in vivo conditions, where the molecules participate in a complex system with multi-faced interactions and feedback mechanisms [161]. It is important that antioxidants be evaluated both alone and in combination to distinguish which specific antioxidant is responsible for certain benefits.

### 2.6. Other Antioxidant Mechanisms

The molecules described in this review have been evaluated for their potential to improve mitochondrial function in reproduction, though many additional molecules may also serve this purpose. Many molecules have antioxidant properties, including growth hormone [162], progesterone [163], and curcumin [164], which successfully reduce oxidative stress in the animal model; only the mechanism of action for growth hormone is suggested to be related to mitochondrial activity improvement [162]. In addition, putrescine supplementation improves oocyte quality and reproductive performance in aged mice [165] and is related to improved mitochondrial activity [166]. Because human granulosa cells from aged follicles present periovulatory putrescine deficiency [167], putrescine supplementation is suggested as a novel therapy to restore human ovarian function. 

Another method of inducing the antioxidant effect is caloric restriction (CR). CR consists of limiting the daily diet to 25–50% of the normal diet. CR extended the lifespan and delayed aging in rodents [168]. In addition, CR can improve fertility and prolong reproductive life by delaying the process of ovarian aging [169]. However, its implementation is not easy in practice, and there are alternative substances that can induce the same effects [170]. For instance, metformin decreases the production of hepatic glucose by inhibiting the mitochondrial ETC complex I; thus, it mimics CR effects while reducing ROS production [171]. 

Other compounds enhance reproductive performance, though their potential antioxidant role has not been evaluated. For example, vitamin D [172] and myo-inositol [173] administration to infertile patients undergoing IVF treatments improved clinical outcomes. The addition of myo-inositol to other molecules with proven antioxidant properties also showed promising results in PCOS [174] and IVF patients [175]. 

In sum, infertility therapy based on antioxidant supplementation is continuously evolving, and new applications of molecules may be adopted to keep oxidative stress in balance. This review covers antioxidants already described in the literature but constitutes only a subset of all molecules with antioxidant properties available. Therefore, continued research in this area is critical to the development of antioxidant therapies and applications.

## 3. Conclusions

Antioxidants are molecules that are easily obtained from natural sources. Their mechanisms of action are diverse, but they typically enhance mitochondrial function or directly scavenge free radicals, which in turn protects mitochondria and other cellular components from oxidative stress. Given the crucial role of mitochondrial activity in oocyte maturation, fertilization, and embryo development, antioxidants may improve ART outcomes by improving oocyte quality.

In ART, antioxidant supplementation can be prescribed as an oral pre-treatment or as an adjuvant in the media during in vitro culture, although the extent of its effects have not been fully elucidated. Indeed, the majority of studies described throughout this review evaluate the indirect consequences of antioxidant supplementation on oocyte quality, evidenced by endpoints such as oocyte maturation, aneuploidy, and pregnancy rates, which may or may not be related to improved mitochondrial function. Although the direct relationship between antioxidant support and improved mitochondrial function is likely, further studies are needed to fully evaluate the consequence of antioxidant treatment on specific mitochondrial parameters, such as mitochondrial membrane potential, morphology, and distribution, as well as oxidative stress markers. In addition, there is no consensus on the optimal dose and duration of treatment, so further evaluation of these parameters is necessary before clinical application of antioxidant strategies. 

Although antioxidant therapy is a promising and safe therapy, well-designed human clinical trials are needed before it is incorporated into routine clinical practice. The population that can benefit from their use must also be clearly defined, and their short- and long-term safety must be evaluated. Further, the mechanisms of each antioxidant’s action at the molecular level and the administration protocol must be clearly defined.

## Figures and Tables

**Table 1:**
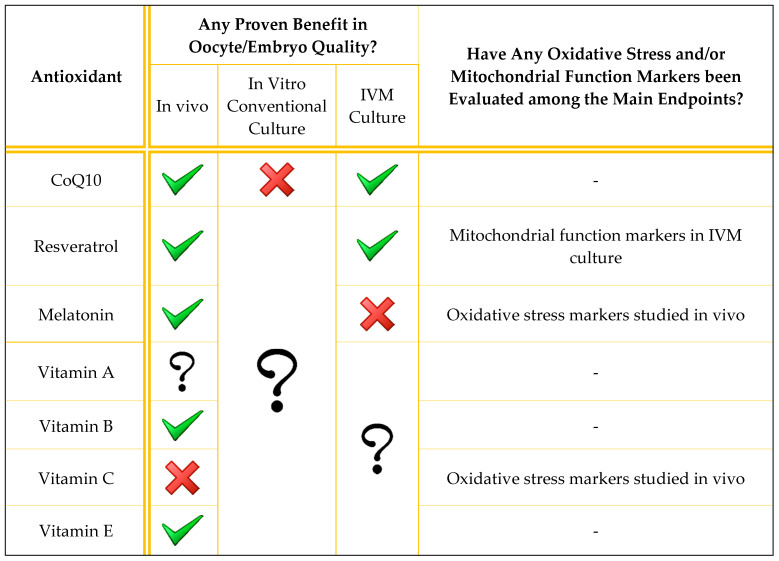
Brief summary of evidence from published studies on the utility of the antioxidants reviewed. Only human studies are summarized. A green tick means that at least one study found beneficial effects on oocyte/embryo quality; a red cross means that the studies reviewed did not find any beneficial effects; a question mark means that the antioxidant effect has not been studied in that scenario.

**Table 2:** Summary of clinical trials conducted to evaluate antioxidant supplementation protocols in vitro and in vivo to improve oocyte quality. *p*-values > 0.05 are presented as *p* = NS (non-significant). Gr.: group. RCT: randomized controlled trial. y.o.: years old. CPR: clinical pregnancy rate. MR: miscarriage rate. LBR: live birth rate. GV: germinal vesicle. OR: odds ratio. CI: confidence interval. IVM: in vitro maturation. hCG: human chorionic gonadotropin. MII: metaphase II. IVF: in vitro fertilization.

Antioxidant	Authors	Type of Study	In Vitro/In Vivo	Intervention	Primary Endpoint	Condition	Treatment Groups (n)	Findings
CoQ10	Bentov et al., 2014 [26]	RCT	In Vivo	600 mg/day for 2 months until the day of oocyte retrieval. Double blinded.	Number of euploid embryos per retrieval	IVF patients 35–43 y.o.	Study gr.: 17 Control gr: 22	Lower aneuploidy rate in the CoQ10 group (46.5% vs. 62.8% in the control group; *p* = NS). Premature termination of the study.
	El Refaeey et al., 2014 [50]	RCT	In Vivo	60 mg three times per day from day 2 of cycle until the day of ovulation induction. Blinded.	Number of follicles >14 and ≥18 mm	CC-resistant PCOS	Study gr.: 51 Control gr.: 50	In the treatment group, higher number of follicles >14 mm (1.94 ± 0.25 vs. 0.13 ± 0.29; *p* < 0.05) and of follicles ≥18 mm (1.85 ± 0.27 vs. 1.30 ± 0.32; *p* < 0.001).
	Xu et al., 2018 [48]	RCT	In Vivo	600 mg/day for 60 days before the initiation of ovarian stimulation	Number of high-quality day-3 embryos	Poor ovarian response patients <35 y.o.	Study gr.: 76 Control gr.: 93	Higher mean number of good quality day-3 embryos in the CoQ10 group (1 vs. 0 in the control group; *p* = 0.03). Secondary endpoints: - Higher ovarian response in the CoQ10 group (4 vs. 2 mean retrieved oocytes; *p* = 0.002). - Higher fertilization rate in the CoQ10 treatment group (67.5% vs. 45.1%; *p* = 0.001). - No significant differences in CPR, MR, and LBR.
	Ma et al., 2020 [53]	RCT	In Vitro	IVM medium supplemented with 50 mmol/L for 24 h	Oocyte maturation and postmeiotic aneuploidy rates	IVM of GV from patients ≥38 y.o. and patients ≤30 y.o.	45 patients ≥38 y.o. Study gr.: 46GV Control gr.: 46GV18 patients ≤30 y.o. Study gr.: 35GV Control gr.: 39GV	Patients ≥38 y.o.: - Higher maturation rate in the CoQ10 group (82.6% vs. 63.0%; *p* = 0.035). - Reduced postmeiotic aneuploidy rate in the CoQ10 group (36.8% vs. 65.5%; *p* = 0.02). Patients ≤30 y.o.: - Similar maturation rates (80.0% in the CoQ10 group vs. 76.9% in the control group; *p* = 0.8). - Similar postmeiotic aneuploidy rates (28.6% in the CoQ10 group vs. 30.0% in the control group; *p* = 0.9).
CoQ10	Kile et al., 2020 [54]	RCT Preliminary results (ASRM Congress 2020)	In Vitro	Mitoquinol addition to the culture media from fertilization and throughout embryo development	Effect on embryo development	Advanced maternal age (≥35 y.o.) women	11 patients Study gr: 66 embryos Control gr: 143 embryos	No differences between control and Mitoquinol treatment in day 5 (18% in control group vs. 20% in the study group) or total (48% vs. 45%) good quality blastocyst development per zygote, total blastocyst development (63% vs. 62%) and euploidy rates (33% vs. 30%); *p* = NS.
Resveratrol	Liu et al., 2018 [68]	RCT	In Vitro	IVM medium supplemented with 1.0 µm for 24 and 36 h	Maturation rates after 24 and 36 h, mitochondrial immunofluores-cence intensity, and proportion of matured oocytes with an abnormal spindle morphology and irregular chromosomal arrangement	IVM of GV from patients 38–45 y.o.	64 patients Study gr.: 38GV Control gr.: 37GV	- Increased maturation rates of the resveratrol group after 24 h (55.3% vs. 37.84% in the control group; *p* < 0.05) and 36 h (71.1% vs. 51.35%; *p* < 0.05) of IVM culture. - Increased mitochondrial immunofluorescence intensity in the resveratrol group (53.0% vs. 31.1%, *p* < 0.05). - Reduced proportion of abnormal spindle morphology and irregular chromosomal arrangement in the resveratrol group (*p* < 0.05).
	Bahramrezaie et al., 2019 [78]	RCT	In Vivo	800 mg/day for 40 days until the day of oocyte retrieval. Triple blinded.	Levels of VEGF expression in granulosa cells	Infertile PCOS patients 18–40 y.o.	Study gr.: 30 Control gr.: 31	- Reduced VEGF expression in the resveratrol group (*p* = 0.0001). Secondary endpoints: - No differences between both groups in the number of mature oocytes and cleavage and fertilization rates (*p* = NS). - Higher high-quality oocyte rate (81.9% vs. 69.1%; *p* = 0.002) and high-quality embryo rate in the resveratrol group (89.8% vs. 78.8%; *p* = 0.024).
Resveratrol	Ochiai et al., 2019 [27]	Retrospective	In Vivo	200 mg/day during the IVF cycle.	Pregnancy outcomes (CPR and MR)	IVF patients	Study gr.: 204 cycles/102 women Control gr.: 7073 cycles/2958 women	Decreased CPR [10.8% vs. 21.5%; *p* = 0.0005 (Adjusted OR 95% CI 0.539, 0.341–0.853] and increased MR [52.4% vs. 21.8%; *p* = 0.0022 (Adjusted OR 95% CI 2.602, 1.070–6.325] after resveratrol supplementation.
Melatonin	Takasaki et al., 2003 [90]	Prospective cohort study with an intrapatient retrospective comparison	In Vivo	1 or 3 mg/day from the fifth day of the previous cycle until the day of ovulation induction	To compare oocyte quality between the previous and current IVF cycles	Women with a previous IVF failure due to poor oocyte quality	Study gr. (1 mg): 13 Control gr.: previous cycle data. Study gr. (3 mg): 23 Control gr.: previous cycle data.	- Reduced number of degenerated oocytes in the 3 mg group (*p* < 0.05) vs. the control group. - Tendency toward an increased fertilization rate in the 3 mg group. - No differences in the numbers of retrieved and mature oocytes between the 3 mg and the control group. - No differences in the number of retrieved, mature, degenerated, and fertilized oocytes between the 1 mg and control group.
	Tamura et al., 2008 [91]	Prospective cohort study with a retrospective comparison in the same population	In Vivo	3 mg/day from the fifth day of the previous cycle until the day of oocyte retrieval	To compare fertilization rates between the previous and current IVF cycles	Women with a previous IVF failure due to low fertilization rate (≤50%)	Study gr.: 56 Control gr.: previous cycle data Placebo gr.: 59 Control gr.: previous cycle data	Increased fertilization rate in the melatonin group (29.8 points compared to the previous IVF cycle; *p* < 0.01), while there were no differences in the placebo group (1.9 points compared to the previous IVF cycle; *p* > 0.01).
	Eryilmaz et al., 2011 [28]	RCT	In Vivo	3 mg/day from the third or fifth day of the previous cycle until the day of oocyte retrieval	Oocyte quality	IVF patients	Study gr.: 30 Control gr.: 30	- Increased number of mature oocytes (9.0 vs. 4.4; *p* = 0.0001) in the treated group. Secondary endpoints: - Increased number of retrieved oocytes (11.5 vs. 6.9; *p* = 0.0001) in the treated group.
	Batıoğlu et al., 2012 [92]	RCT	In Vivo	3 mg/day during the IVF cycle	Number of MII oocytes	IVF patients	Study gr.: 40 Control gr.: 45	- No differences in the mean number of MII oocytes retrieved (12.0 in the study vs. 10.9 in the control gr.; *p* = 0.139). - Higher percentage of MII oocytes/retrieved in the treated group (81.9% in the study vs. 75.8% in the control gr.; *p* = 0.034).
Melatonin	Kim et al., 2013 [100]	RCT	In Vitro	IVM medium supplemented with 10 µmol/L for 24 and 48 h	Maturation rates	IVM of GV from PCOS patients with or without hCG priming during unstimula-ted cycles	Study gr.: 62 (41 non-hCG primed, 21 hCG primed) Control gr.: 49 (25 non-hCG primed, 24 hCG primed)	- In the non-hCG priming gr., there were no differences in the maturation rate between melatonin treatment and the control gr. after 24 h (40.0% vs. 40.0%; *p* = NS) and 48 h (62.5% vs. 60.3%; *p* = NS) of maturation. - In the hCG priming gr., there were no differences in the maturation rate between melatonin treatment and control gr. after 24 h (51.3% vs. 44.9%; *p* = NS) and 48 h (59.8% vs. 54.8%; *p* = NS) of maturation.
	Fernando et al., 2018 [96]	RCT	In Vivo	2/4/8 mg/twice per day during ovarian stimulation. Double blind.	CPR	IVF patients	Study gr. (2 mg): 41 Study gr. (4 mg): 39 Study gr. (8 mg): 40 Control gr.: 40	- No differences in CPR (15% in the control group vs. 26.8 in the 2 mg group; 15.4 in the 4 mg group; 22.5 in the 8 mg group; *p* = 0.5). Secondary endpoints: - No differences in the total oocyte number (*p* = 0.8), number of MII oocytes (*p* = 0.4), number of fertilized oocytes (*p* = 0.6) and the number (*p* = 0.6) or quality (*p* = 0.9) of embryos between any of the three treatment groups and the control group.
	Espino et al., 2019 [97]	RCT	In Vivo	3 or 6 mg/day from the onset of ovarian stimulation until the day of oocyte retrieval	- TAC, SOD, and LPO as antioxidant markers in the follicular fluid. - 8-OHdG as oxidative stress marker in the follicular fluid. - IVF success.	IVF patients with unexplained infertility	Study gr. (placebo): 10 Study gr. (3 mg): 10 Study gr. (6 mg): 10 Non-randomized control gr. (fertile women): 10	- Restored concentrations of TAC, SOD, LPO, and 8-OHdG in the follicular fluid of the study gr. to levels found in fertile women (except for SOD levels in the 3 mg study gr.). - Improved MII oocyte rate (83.6% in the 3 mg gr. vs. 81.9 in the fertile gr., *p* = NS, and in comparison to 70.6 in the no-treatment gr., *p* < 0.05). - Improved MII oocyte rate (76.2% in the 6 mg gr., vs. 81.9% in the fertile gr. and 70.6% in the no-treatment gr.; *p* = NS). - Increased number of transferable embryos (5.1 in the 3 mg gr. and 4.6 in the 6 mg gr.; vs. 2.3 in the fertile gr., *p* < 0.05, and 2.0 in the no-treatment gr., *p* < 0.05).
Folic acid	Gaskins et al., 2014 [138]	Prospective cohort	In Vivo	Validated food frequency questionnaire, with specific data about supplemental folic acid intake	To assess the relationship between pregnancy outcomes (implantation rate, CPR, and LBR) and supplemental folate intake	Infertile IVF patients	Total n: 232 Quartile 1 (Q1) Folic acid <400 µg/day): 51 Quartile 4 (Q4) Folic acid >800 µg/day): 60	- Higher implantation rates [Adjusted mean; A.m., 95% CI 0.67 (0.56, 0.77) vs. 0.43 (0.31, 0.55)], CPR [A.m., 95% CI 0.62 (0.51, 0.73) vs. 0.41 (0.29, 0.53)], and LBR [A.m., 95% CI 0.55 (0.43, 0.66) vs. 0.35 (0.24, 0.48)], in the Q4 in comparison to Q1 (*p* < 0.05). - Positive linear relationship between supplemental folate and LBR up to 1200 µg/day, without evidence of additional benefit with higher intakes.
	Murto et al., 2014 [140]	Longitudinal cohort study	In Vivo	Serum folate determinations (folate status) and folic acid supplement questionnaires (folic acid intake)	CPR and LBR	IVF patients with unexplained infertility	Total n: 180 [Serum folate] ≥22.5 nmol/L: 78/180<22.5 nmol/L: 89/180 No data: 13 Folic acid supplements intake Users: 137/180 Non-users: 42/180 No data: 1/180	Folate status: - No statistically significant differences regarding CPR [35.9% when serum folate ≥22.5 nmol/L vs. 34.8%; OR 95% CI 0.954 (0.505–1.802)] and LBR [28.2% when serum folate ≥22.5 nmol/L vs. 27.0%; OR 95% CI 0.940 (0.476–1.855)]. Folic acid intake: - No statistically significant differences regarding CPR [32.8% users vs. 35.7% non-users; OR 95% CI 1.003 (0.515–1.953)] and LBR [24.1% users vs. 31.0% non-users; OR 95% CI 1.366 (0.677–2.757)].
Ascorbic acid	Griesinger et al., 2002 [145]	RCT	In Vivo	1/5/10 g/day from the day of oocyte retrieval and during the luteal phase (14 days). Double blind.	Implantation rate and CPR	Infertile IVF patients	Study group (1g): 172 Study group (5g): 153 Study group (10g): 136 Control gr.:158	- Implantation rate was 10.0% in the 1 g group, 12.36% in the 5 g group, 10.3% in the 10 g group, and 14.8% in the control group (*p* = 0.186). - CPR was 22% in the 1 g group, 24% in the 5 g group, 21% in the 10 g group, and 28% in the control group (*p* = 0.186).
Ascorbic acid	Crha et al., 2003 [144]	Prospective cohort	In Vivo	500 mg/day during ovarian stimulation	Number of pregnancies	Infertile IVF patients	Study gr.: 38 Control gr.: 38	No significant difference in the number of pregnancies (34.2% vs. 23.7% in the control group; *p* = NS).
	Lu et al., 2018 [146]	RCT	In Vivo	1000 mg/day from 2 months before IVF treatment until 2 weeks after embryo transfer	- Serum and follicular fluid levels of ascorbic acid. - Levels of oxidative stress markers	Endometriosis patients	Study gr.: 137 Placebo gr.: 108	- Higher serum and follicular fluid levels of ascorbic acid (levels not shown; *p* < 0.05) in the study group. - No difference in oxidative stress markers after treatment.
Vitamin E	Bahadori et al., 2017 [155]	Observational	In Vivo	Serum and follicular fluid vitamin E determination	To assess the relationship between serum and follicular fluid vitamin E levels and oocyte maturation and embryo quality	IVF patients with a history of vitamin E supplementa-tion	Total n: 50 Follicular fluid ranges (mg/dL) 0.35–1 1–1.5 1.5–2 2–7.4 Serum ranges (mg/dL) 1-5 5-10 10–15 15–0	Follicular fluid: - Vitamin E levels within the ranges of 0.35–1 mg/dL and 1.5–2 mg/dL were related to higher oocyte maturation rates (89.2% and 84.9%, respectively, vs. 69.6% in 1–1.5 mg/dL and 76.7% in 2–7.4 mg/dL ranges; *p* = 0.002). - No significant relationship between vitamin E levels and embryo quality was observed. Serum: - Vitamin E levels between 10 and 15 mg/dL were correlated with a higher proportion of high-quality embryos (87.5% vs. 46.2 in 1-5 mg/dL, 54.9% in 5–10 mg/dL, 42.9% in 15-20 mg/dL; *p* = 0.007). - No significant relationship between vitamin E levels and oocyte maturation was observed. A higher proportion of MII oocytes in women with vitamin E supplementation (87.4% vs.77% in women without supplementation; *p* = 0.010).
Antioxidants in combination	Fatemi et al., 2017 [156]	RCT	In Vivo	Vitamin E (400 mg/day) and Vitamin D (50,000 IU/one in two weeks) for 8 weeks. Double blinded.	Implantation rate, pregnancy rate, and CPR	PCOS infertile women	Study gr.: 44 Control gr.: 46	Higher implantation (35.1% vs. 8.6%; *p* < 0.001), pregnancy (69.0% vs. 25.8%; *p* < 0.001), and CPR (62.1% vs. 22.6%; *p* = 0.002) in the treated group.
	Ozkaya et al., 2011 [160]	RCT	In Vivo	Vitamins A, B, C, D, E, H; calcium; folic acid; iron; nicotinic acid; magnesium; phosphor; copper; manganese; zinc For 45 days before serum and follicular fluid collection	Follicular fluid and serum antioxidant capacity	IVF patients	Study gr.: 26Placebo gr.: 30	- Higher serum and follicular fluid antioxidant capacity were observed in the treated group. - Higher serum vitamins C (61.6 µmol/L vs. 57.9 µmol/L in the control group; *p* < 0.05) and A (2.3 µmol/L vs. 1.5 µmol/L; *p* < 0.01); and higher follicular fluid glutathione (0.4 µmol/L vs. 0.2 µmol/L; *p* < 0.01) and vitamin C (84.5 µmol/L vs. 52.7 µmol/L; *p* < 0.01) and E (8.3 µmol/L vs. 5.0 µmol/L; *p* < 0.001) concentrations.
	Youssef et al., 2014 [159]	RCT	In Vivo	Vitamins A, E, C Zinc Molybdenum Selenium Biotin Bioflavonoid	Number of MII oocytes	IVF patients with unexplained infertility	Study group: 112 Control group: 106	No difference in the mean number of MII oocytes between the treated (12.7) and the control group (13.2); *p* = 0.7.
